# Change in the serum selenium level of patients with non-metastatic and metastatic non-small cell lung cancer (NSCLC) during radiotherapy as a predictive factor for survival

**DOI:** 10.1007/s00066-024-02276-w

**Published:** 2024-09-06

**Authors:** Julia Ohlinger, Dirk Vordermark, Christian Ostheimer, Matthias Bache, Therese Tzschoppe, Kamil Demircan, Lutz Schomburg, Daniel Medenwald, Barbara Seliger

**Affiliations:** 1 Medical Faculty, Radiation Therapy Clinic, University Hospital Halle (Saale), Martin Luther University Halle-Wittenberg, Ernst-Grube-Straße 40, 06120 Halle (Saale), Germany; 2 Charité—University Medicine Berlin, Institute for Experimental Endocrinology, Berlin, Germany; 3 Medical Faculty, Martin-Luther-University Halle-Wittenberg, Halle, Germany; 4 Institute for Translational Immunology, Brandenburg Medical School “Theodor Fontane”, Brandenburg, Germany; 5 Fraunhofer Institute for Cell Therapy and Immunology, Leipzig, Germany

**Keywords:** Radiation, Nutrition, Chemotherapy, Overall survival, Immunotherapy

## Abstract

**Background:**

Lung cancer remains a serious medical problem. The trace element selenium seems to be a promising prognostic marker or therapeutic option for cancer patients.

**Methods:**

We enrolled 99 patients with histologically confirmed NSCLC undergoing radiotherapy. The serum selenium level of these patients was determined prior to irradiation (t0), after reaching 20 Gy (t1), and at the end of radiotherapy (t2). Selenium concentrations were measured with total-reflection X‑ray fluorescence (TXRF) spectroscopy. We formed three subgroups according to the change in serum selenium levels across timepoints, and Kaplan–Meier analysis was used to estimate overall survival (OS). Further subgroups were patients with/without metastatic disease. We used adjusted Cox regression models.

**Results:**

The change in selenium concentration was especially significant between t0 and t1 for the whole study group (hazard ratio [HR] = 0.5, *p* = 0.03) as well as in patients with metastasized NSCLC (HR = 0.3, *p* = 0.04) after adjustment. The baseline selenium value in patients with non-metastasized NSCLC was associated with overall survival (HR = 0.3, *p* = 0.04). The change in selenium levels between t0 and t2 was significant in patients with metastatic lung cancer (HR = 0.1, *p* = 0.03). Patients with increased serum selenium levels during radiotherapy between the start of treatment (t0) and t1 had better OS (HR = 0.46, *p* = 0.05).

**Conclusion:**

Especially patients with increasing selenium levels during radiotherapy showed an improved overall survival. Thus, serum selenium might be a predictive factor for OS in NSCLC patients. The value of supplementation of the trace element is subject to future research.

## Introduction

Cancer in general is the third most frequent cause of death worldwide and thus a serious medical problem, with lung cancer being one of the most commonly diagnosed cancers [1]. Lung cancer can be categorized into non-small cell lung cancer (NSCLC; around 85%) and small cell lung cancer (SCLC; 15%). Most patients are diagnosed in an advanced stage of disease, which limits the therapeutic options and overall survival (OS) of patients. Nearly 50% of all cancer patients undergo irradiation during their treatment course [2]. Despite an improvement in the efficacy of standard treatments like chemotherapy, surgery, tyrosine kinase inhibitors (TKI), and recently also immunotherapy [2–6], lung cancer remains the leading cause of cancer deaths even in highly developed countries [1]. So far, not all lung cancer patients benefit from treatment at every stage of the disease. The development of resistance, adverse events, and unavoidable disease progression highlights the urgent need for novel diagnostic, prognostic, and therapeutic options for these diseases. To this end, reliable biomarkers that predict disease course and help to stratify patients in terms of those who are likely to experience a clinical benefit from certain therapies are urgently needed [7]. Regarding prognostic factors, cancer patients tend to have lower serum selenium levels than the general population [8, 9], making selenium a potential prognostic marker or therapeutic option. The importance of selenium for the antioxidant defense system and the immune response has been reported in many studies. Incorporated into proteins as selenocysteine, it influences the activity of a number of essential selenoproteins, such as members of the thioredoxin reductase, glutathione peroxidase, and iodothyronine deiodinase families, as well as many selenoproteins which directly affect the inflammatory response, apoptosis of cells, and status of reactive oxygen species (ROS) [10, 11]. Although the correlations between selenium status, cancer risk, and radiotherapy have been studied over the years, the results are heterogeneous, and underlying mechanisms of function remain unknown. Concerning lung cancer patients, a systematic review and meta-analysis demonstrated that selenium may be effective for lung cancer prevention and may reduce side effects of radiation. In addition, many in vitro studies have investigated the effect of selenium in irradiated breast cancer cell lines [12], normal as well as malignant human mononuclear blood cells [13], and lung cancer cells [14]. An increased cytotoxic effect of radiotherapy in malignant cells was observed during selenium substitution. In addition, dose-limiting side effects of anticancer treatments appear to be reduced in vitro. In vivo studies provide evidence of a benefit of selenium treatment during irradiation [15]. Therefore, this study aims to assess the serum selenium status of patients and correlates the results to survival in patients with histologically confirmed NSCLC prior to and during radiotherapy.

## Materials and methods

### Study participants

**Fig. 1:**
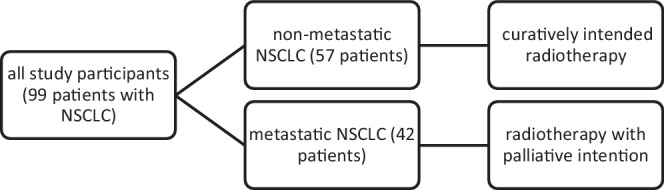
Overview of study participants. *NSCLC* non-small cell lung cancer

From May 2017 to August 2020, 99 patients with histologically confirmed NSCLC undergoing radiotherapy at the Martin Luther University Halle-Wittenberg, Halle, Department of Radiation Oncology, were prospectively enrolled (see Fig. 1). The inclusion criteria for participation in this study consisted of (i) age ≥ 18 years, (ii) histologically confirmed NSCLC without further treatments, and (iii) no other diagnosed cancers in the past 5 years. All participants gave written informed consent. We classified the tumor stage according to the Union for International Cancer Control (UICC) classification of malignant tumors. Blood samples of patients were collected prior to irradiation (t0), after reaching 20 Gy (t1), and at the end of radiotherapy (t2). The first follow-up of patients was 4 up to 6 weeks after the end of radiotherapy. Survival status was obtained from the local citizen registration offices or regular record, if appropriate. The Ethics Committee of the Medical Faculty of the Martin Luther University approved the study (no.: 2017-15).

### Determination of selenium levels

Serum samples were analyzed for selenium concentrations by total-reflection X‑ray fluorescence (TXRF) spectroscopy. To this end, aliquots of the samples were spiked with a gallium solution for standardization, applied to polished glass slides, and dried. Excitation by X‑ray was conducted in a TXRF spectrometer (S4 T‑STAR, Bruker nano GmbH, Berlin, Germany) and selenium concentrations were determined from the areas under the curve of the emission spectra, as described in [16, 17].

### Statistical analysis

The Cox proportional hazard regression model was used to estimate hazard ratios (HR) and respective 95% confidence intervals (CIs) for all univariate and multivariate analyses. All study participants and the subgroups of patients with metastatic and non-metastatic NSCLC were subjected to analyses of different parameters in terms of timepoints (t0 before the start of irradiation, t1 after reaching 20 Gy, and t2 at the end of radiation), sex, age, stage of disease (whether the tumor was metastasized or not), and the biological equivalent dose (for 2 Gy called EQD2). In addition, the patient groups were separated into three subgroups according to the serum selenium levels across timepoints: patients with the highest increase (3), patients with a limited change in selenium (2), and participants with the strongest decrease in the serum selenium level between different points in time (1). The standard error of the change in selenium levels was used to distinguish between these three subgroups. Therefore, the limit was placed at ±8.5 μg/l for the change between t0 and t1 and at ±11.4 μg/l for the alteration between t0 and t2. Furthermore, Kaplan–Meier analysis was used to estimate overall survival (OS) in the patient subgroups. All statistical analyses were performed using IBM SPSS Statistics (version 28; IBM Corp., Armonk, NY, USA). Statistical *p*-values < 0.05 were statistically significant.

## Results

### Features of the study cohort

Regarding treatment of the study cohort, 57 patients with non-metastatic NSCLC received curatively intended radiotherapy (2 Gy/day, 5 fractions/week, to a total dose of 66 Gy) and 42 patients with metastasized disease were irradiated with palliative intent (3 Gy/day, 5 fractions/week, to a total dose of at least 36 Gy) as shown in Fig. 1. In addition, most patients were also subjected to chemotherapy using combinations of different cytostatic drugs, such as carboplatin, cisplatin, gemcitabine, docetaxel, paclitaxel, or vinorelbine.

As shown in Table 1, the mean age of NSCLC patients was 66.8 years (range 46–88 years), with 70% male study participants and 96% smokers. Median OS, defined as death or last seen as an outpatient in the Department of Radiotherapy of the University Hospital of Halle, Germany, was 10 months after the end of therapy. Prior chemotherapy had been received by 57% of patients. Ten participants received stereotactic irradiation. As shown in Table 2, all patients with metastasized disease had a smoking history, and 93% of patients with a non-metastatic tumor were smokers. Median OS differed between the subgroups: patients with metastatic lung cancer survived 3.8 months, while those with non-metastatic disease had a median OS of about 16.1 months.

**Table 1:** Characteristics of the whole study group (*n* = 99)

	*N*	%
*Sex*		
Male	70	70.7
Female	29	29.3
*Age, mean (range) in years*	66.8 (46–88)	–
*Smoking status*		
Yes	95	96.0
No	4	4.0
*Presence of metastases*		
Non-metastatic	57	57.6
Metastatic	42	42.4
*Chemotherapy*		
Yes	56	56.6
No	43	43.4
*Stereotactic*		
Yes	10	10.1
No	89	89.9
*EQD2, mean (range) in Gy*	61.9 (31.3–190.9)	–
*Borders of Kaplan–Meier analysis between t0 and t1*		
(1) most decrease (≤ −8.5 μg/l)	14	14.1
(2) almost no change (−8.5–+8.5 μg/l)	49	49.5
(3) most increase (≥ +8.5 μg/l)	36	36.4
*Borders of Kaplan-Meier analysis between t0 and t2*		
(1) most decrease (≤ −11.4 μg/l)	16	16.2
(2) almost no change (−11.4–+11.4 μg/l)	52	52.5
(3) most increase (≥ +11.4 μg/l)	31	31.3
*Overall survival (median) in months*	10	–
*Serum selenium level (mean) in μg/l*		
t0	63.6 (±18.5)	–
t1	83.1 (±45.3)	–
t2	83.6 (±59.0)	–

**Table 2:** Comparison of patient features between non-metastatic (*n* = 57) and metastatic NSCLC (*n* = 42)

	Non-metastatic (*n* = 57)	Metastatic (*n* = 42)
*Sex*		
Male	42 (73.7%)	28 (66.7%)
Female	15 (26.3%)	14 (33.3%)
*Mean age in years*	68.8	64.5
*Smoking*	53 (93.0%)	42 (100%)
*OS median in months*	16.1	3.8
*EQD2, mean in Gy*	62.5	52.1
*Serum selenium levels (mean) in μg/l*		
t0	62.4	64.9
t1	94.9	67.9
t2	97.9	65.9

### Treatment-induced alterations of serum selenium levels

**Fig. 2:**
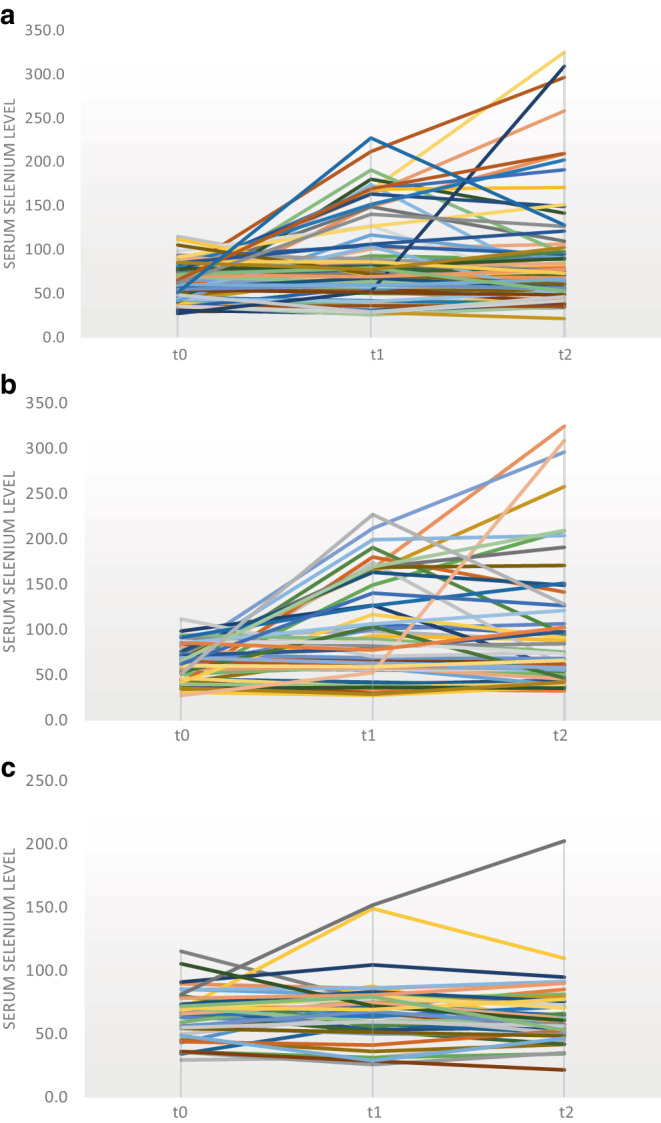
**a** Change in serum selenium levels of all non-small cell lung cancer (NSCLC) patients (*n* = 99) over time. The serum selenium levels were determined prior to radiotherapy (t0), after reaching 20 Gy (t1), and at the end of radiotherapy (t2). The data are presented as serum selenium levels in μg/l over time. **b** Change in serum selenium levels of all patients with non-metastatic NSCLC (*n* = 57) over time. **c** Change in serum selenium levels of all patients with metastatic NSCLC (*n* = 42) over time

While in non-metastatic NSCLC patients the serum selenium levels significantly increased from 62.4 μg/l to 94.9 μg/l at t1 and 97.9 μg/l at t2, no or only a marginal increase was found at t1 and t2 vs. t0 in metastatic NSCLC patients. However, it is noteworthy that the increase in the serum selenium levels was highly variable over time upon treatment of non-metastatic NSCLC patients (Fig. 2b,c). Moreover, all patients who had undergone additional chemotherapy were analyzed. This subgroup comprised 55 patients, but none of the parameters analyzed were significantly associated with overall survival (Table 6 of the appendix).

Prior to radiotherapy (t0), the mean serum level of selenium in all patients was 63.6 μg/l. After reaching 20 Gy (t1), the mean selenium levels increased to 83.1 μg/l and to 83.6 μg/l at the end of radiotherapy (t2; Fig. 2a). Moreover, comparison of the selenium levels of non-metastatic vs. metastatic NSCLC patients demonstrated differences in the selenium values of patients after radiotherapy (Table 2, Fig. 2b,c). In contrast, other investigated factors seemed to be almost comparable between both subgroups.

### Correlation of the serum selenium status with clinical parameters of NSCLC patients at the distinct timepoints

For all study participants (*n* = 99), the results of the univariate and multivariate analyses of selenium status are presented in Table 3. At t0 and t1, the selenium value at t1 was significant in terms of overall survival with a hazard ratio of 0.48 (CI 0.3–0.8; *p* = 0.005) for the univariate analysis. The selenium value at t2 was only significant in the univariate analysis with a HR of 0.6 (CI 0.3–0.95; *p* = 0.03). For the multivariate analyses, five models were investigated by adjusting serum selenium levels for t1/t2 (model I); t1/t2 and age (model II); t1/t2, age, and sex (model III); t1/t2, age, sex, and presence of metastasis (non-metastatic reference, metastatic value; model IV); and t1/t2, age, sex, presence of metastasis (non-metastatic reference, metastatic value), and EQD2 (model V). A significant hazard ratio (HR) of 0.5 (CI 0.3–0.9; *p* = 0.03) was found in models I, II, and III for the multivariate analysis between timepoints t0 and t1.

**Table 3:** Univariate and multivariate analysis of selenium levels in the study cohort (*n* = 99) at timepoints t0 and t1 and timepoints t0 and t2

	Univariate analysis			Multivariate analysis														
				Model 1 (I^a^)			Model 2 (II^b^)			Model 3 (III^c^)			Model 4 (IV^d^)			Model 5 (V^e^)		
	HR	95% CI	*p*-value	HR	95% CI	*p*-value	HR	95% CI	*p*-value	HR	95% CI	*p*-value	HR	95% CI	*p*-value	HR	95% CI	*p*-value
t0	0.5	0.2–1.1	0.096	0.9	0.3–2.5	0.9	0.92	0.3–2.5	0.9	0.99	0.4–2.7	0.99	0.6	0.2–1.8	0.4	0.6	0.2–1.7	0.3
t1	0.48	0.3–0.8	0.005	0.5	0.3–0.9	0.03	0.5	0.3–0.9	0.03	0.5	0.3–0.9	0.03	0.6	0.3–1.2	0.2	0.6	0.3–1.2	0.2
t2	0.6	0.3–0.95	0.03	0.3	0.4–1.1	0.09	0.6	0.4–1.1	0.09	0.6	0.3–1.1	0.08	0.7	0.4–1.4	0.4	0.7	0.4–1.4	0.4

Hazard ratios (HR), 95% confidence intervals (CI), and *p*-values of overall survival adjusted for serum levels at baseline prior to, during, and at the end of therapy; age; gender; tumor staging/metastasis formation, and EQD2 are presented in the whole study cohort

^a^I: adjusted for t1/t2

^b^II: adjusted for t1/t2 and age

^c^III: adjusted for t1/t2, age, and sex

^d^IV: adjusted for t1/t2, age, sex, and presence of metastasis (non-metastatic reference, metastatic value)

^e^V: adjusted for t1/t2, age, sex, presence of metastasis (non-metastatic reference, metastatic value), and EQD2

**Fig. 3:**
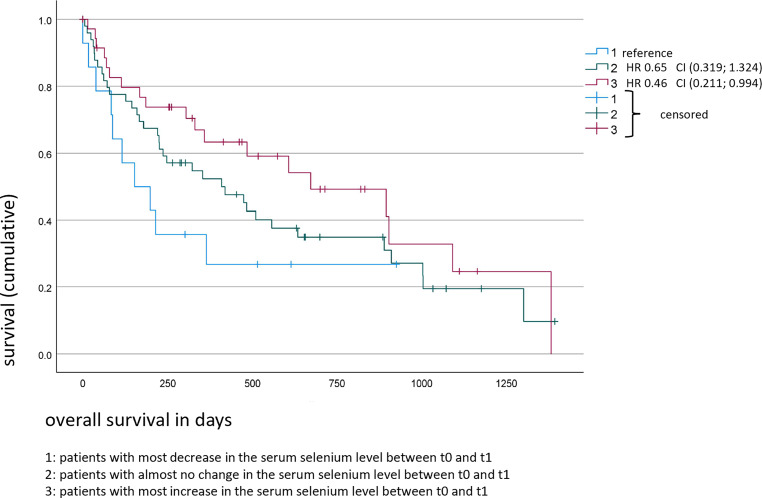
Overall survival of all non-small cell lung cancer (NSCLC) patients (*n* = 99) depending on the trend in serum selenium levels between timepoints t0 and t1

**Fig. 4:**
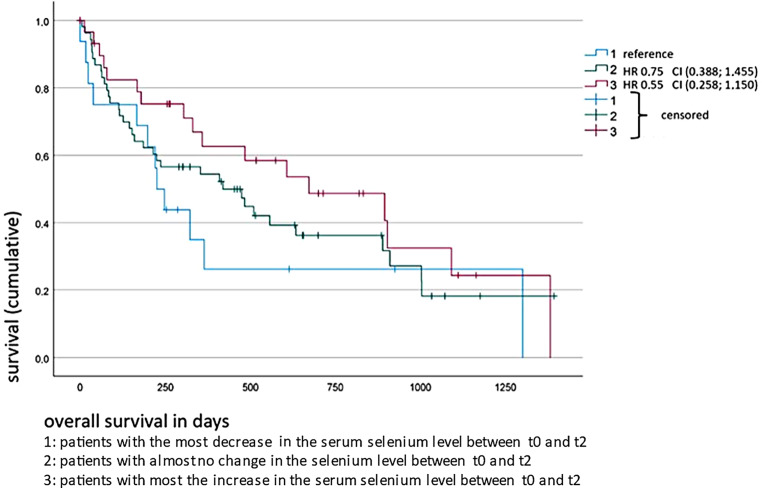
Overall survival of all non-small cell lung cancer (NSCLC) patients (*n* = 99) depending on the trend in serum selenium levels between timepoints t0 and t2

Moreover, the dependence of OS on the change in serum selenium levels between t0 and t1/t2 was analyzed via the Kaplan–Meier method (Figs. 3 and 4). The patients with the highest increase in serum selenium values from t0 to t1 had a significantly higher OS rate (HR 0.46, CI 0.2–0.99; *p* = 0.05) than the study participants without any change or with a decrease in selenium levels. Analyses of the changes in serum selenium concentrations from t0 to t2 had the same effect in patients with the highest increase, but this was not significantly associated with their overall survival (HR 0.55, CI 0.3–1.2; *p* = 0.1).

As shown in Table 4, baseline selenium values at t0 were only significant in univariate models (HR 0.3, CI 0.1–0.9; *p* = 0.04) in relation to overall survival in all non-metastatic NSCLC patients (*n* = 57). In contrast, no significant results for other adjusted factors were obtained.

**Table 4:** Univariate and multivariate analysis of selenium levels in patients with non-metastatic non-small cell lung cancer (NSCLC; *n* = 57) over time

	Univariate analysis			Multivariate analysis											
				Model 1 (I^a^)			Model 2 (II^b^)			Model 3 (III^c^)			Model 4 (IV^d^)		
	HR	95% CI	*p*-value	HR	95% CI	*p*-value	HR	95% CI	*p*-value	HR	95% CI	*p*-value	HR	95% CI	*p*-value
t0	0.3	0.1–0.9	0.04	0.4	0.1–1.3	0.1	0.4	0.1–1.4	0.2	0.5	0.1–2.0	0.3	0.4	0.1–1.6	0.2
t1	0.6	0.3–1.2	0.2	0.8	0.4–1.8	0.6	0.8	0.4–1.8	0.7	0.8	0.4–1.6	0.5	0.7	0.3‑1.5	0.4
t2	0.8	0.4–1.5	0.4	0.95	0.5–1.7	0.9	0.97	0.5–1.8	0.9	0.95	0.5–1.8	0.9	0.9	0.5–1.7	0.8

Hazard ratios (*HR*), 95% confidence intervals (*CI*), and *p*-values of overall survival adjusted for serum levels at baseline prior to, during, and at the end of therapy; age; gender; and EQD2 are presented in patients with non-metastatic NSCLC

^a^I: adjusted for t1/t2

^b^II: adjusted for t1/t2 and age

^c^III: adjusted for t1/t2, age and sex

^d^IV: adjusted for t1/t2, age, sex and EQD2

Regarding the metastasized NSCLC patients (*n* = 42) analyzed at t0 and t1, the serum selenium value at t1 was significantly associated with the patients’ overall survival in all analyzed models with the exception of model II (Table 5). In addition, the serum selenium level of t2 seemed to be significant in the univariate analysis with an HR of 0.2 (CI 0.1–0.8; *p* = 0.02; Table 5) and also in all adjusted models (models I–IV) of multivariate investigation with a hazard ratio of 0.1 (CI 0.02–0.8; *p* = 0.03).

**Table 5:** Univariate and multivariate analysis of selenium levels in patients with metastatic non-small cell lung cancer (NSCLC;* n* = 42) over time

	Univariate analysis			Multivariate analysis											
				Model 1 (I^a^)			Model 2 (II^b^)			Model 3 (III^c^)			Model 4 (IV^d^)		
	HR	95% CI	*p*-value	HR	95% CI	*p*-value	HR	95% CI	*p*-value	HR	95% CI	*p*-value	HR	95% CI	*p*-value
t0	0.7	0.2–2.5	0.5	2.2	0.4–13.3	0.4	1.7	0.2–12.2	0.6	2.0	0.3–15.5	0.5	2.0	0.3–15.9	0.5
t1	0.4	0.2–0.97	0.04	0.3	0.1–0.96	0.04	0.3	0.1–1.05	0.06	0.2	0.04–0.95	0.04	0.2	0.04–0.95	0.04
t2	0.2	0.1–0.8	0.02	0.1	0.02–0.6	0.01	0.1	0.02–0.8	0.03	0.1	0.02–0.8	0.03	0.1	0.02–0.8	0.03

Hazard ratios (HR), 95% confidence intervals and *p*-values of OS adjusted for serum levels at baseline prior to, during, and at the end of therapy; age; gender; and EQD2 are presented in patients with metastatic NSCLC

^a^I: adjusted for t1/t2

^b^II: adjusted for t1/t2 and age

^c^III: adjusted for t1/t2, age and sex

^d^IV: adjusted for t1/t2, age, sex and EQD2

### Features of the metastatic disease cohort

The investigated cohort of 42 patients in total with metastatic disease comprised cases with one (*n* = 3) to five affected sites (*n* = 4). In detail, intrapulmonary metastases (*n* = 33) were the most frequent localization followed by bone metastases (*n* = 24). SBRT was used in five cases, while the majority received hypofractioned radiotherapy with single doses of 2.5 Gy or higher (*n* = 36). In this cohort, 23 patients received some form of thoracic radiation, with the thorax being the most frequently treated region.

The group of patients with metastatic disease showed metastases at several sites, which suggests that they had a considerably higher tumor burden than the patients with non-metastatic disease.

## Discussion

In this study, we describe significant associations of relative selenium deficiency and decline in selenium status with shorter survival odds in lung cancer. A proper supply of micronutrients, such as selenium, is essential for an efficient immune response, thereby reducing the risk of cancer incidence, fast progression, and adverse therapeutic effects. So far, little information exists about serum selenium levels upon radiotherapy. This study demonstrates the prognostic value of assessing changes in serum selenium levels for the first time. The selenium status at t0 was comparable between all groups and only patients with metastasized disease had almost no change during and after irradiation. The change in selenium levels between the start of treatment (t0) and the first timepoint after treatment (t1) was statistically significant in terms of the patients’ overall survival for the whole study cohort (models I–III); likewise, the patients with increased serum selenium levels during radiotherapy had the best OS (Fig. 3). In addition, significant associations with OS for the baseline selenium value t0 (univariate analysis) in patients with non-metastasized NSCLC were found. The changes in serum selenium between t0 and t1 (models I, III, IV) and also between t0 and t2 (models I–IV) were significant in terms of overall survival for metastatic cancer patients. To our knowledge, this is the first study to report altered selenium levels in lung cancer patients at different timepoints of radiotherapy and their association with the OS of patients.

The mean baseline serum selenium level of the study collective analyzed is lower when compared to healthy persons as references, e.g., from the participants of the EPIC cohort enrolled in Potsdam, Germany (median serum Se 80.0 µg/L, interquartile range 19.1 µg/L) [18]. A correlation between low serum selenium levels and cancer mortality has been reported in many entities, including liver, colorectal, and breast cancer [17, 19–21]. Low serum selenium concentrations prior to therapy in stage I NSCLC patients were associated with decreased OS [22]. In addition, Lubiński and coauthors described very low selenium levels in laryngeal cancer patients at the time of diagnosis, which correlated with tumor progression and an increased risk of death [23]. Thus, serum selenium levels were of prognostic relevance prior to the initiation of anticancer treatment, especially in advanced stages of laryngeal tumors [23]. Due to malnutrition caused by invasive surgery and extended radiotherapy, also head and neck cancer patients have a lower selenium status [24]. The published reports confirm our results, since the patients of our study cohort with the lowest selenium levels between t0 and t1 or t0 and t2 had reduced OS. So far, the underlying mechanisms explaining why some cancer patients tend to have lower selenium levels have not yet been identified. However, it has been suggested that malignant disease might be associated with a low selenium status as a consequence of modified metabolism in cancer cells due to tumor-associated inflammation and reduced selenoprotein biosynthesis in the liver or due to predisposition [25, 26].

Moreover, other studies investigating the effect of irradiation on the selenium status in cancer patients including breast cancer patients reported decreased selenium concentrations in patients undergoing radiotherapy [27–29]. In contrast, no change in selenium levels was detected in other studies [24, 30]. Importantly, a study in gynecological tumors indicated that supplemental selenium can be applied as adjuvant treatment in order to reduce the side effects of radiotherapy, without obvious effects on efficacy [31–33]. However, these studies are not comparable to our investigation as the irradiated body area was much larger and the patients received other treatments prior to radiotherapy, or the authors assessed a different tumor entity. Zeng et al. analyzed NSCLC patients with brain metastases and found decreasing selenium levels [29]. Despite the same tumor entity being investigated, this study is different due to the distinct metabolism of the brain. In our study, the mean serum selenium concentration increased during therapy in the whole study cohort, in particular in patients with non-metastatic cancer, but only slightly in metastasized tumor patients. The underlying mechanisms causing the increased selenium levels in this subgroup have not been identified. The in vitro analysis of Chen and coauthors described a potentially antimetastatic influence of selenium on lung cancer cells [34]. Tian and coauthors investigated the effect of selenium nanoparticles on NSCLC cells during irradiation and found a decrease in cell migration and cell invasion and an increase in apoptosis of lung cancer cells [14]. These data suggest that selenium may reduce progression of the malignant disease and, in the case of our investigated study cohort, increase OS.

Regarding the limitations of our study, the German local citizen registration offices do not provide information about the cause of death, so lung cancer-specific mortality could not be estimated. The majority of patients presumably died as a result of their severe malignant lung disease. In addition, smoking tobacco can influence the selenium status [35]. However, this bias can be reduced to a minimum extent because more than 90% of our study participants had smoking habits. We analyzed the selenium value after the diagnosis of cancer and during the limited period of radiotherapy, so the impact of genetic and epigenetic factors is negligible during this short timespan. In addition, nutrition plays an important role in the serum status of trace elements. However, the participants of our study neither received oral nor parenteral selenium substitution. Moreover, most patients stayed in the hospital during the time of irradiation and thus received no additional selenium intake. In conclusion, selenium levels could be influenced by the malignant disease, as already considered by Lopez-Saez and coauthors [25], or deficits may have pre-existed as a risk factor for lung cancer. Our study extended this knowledge by investigating whether changes in selenium levels during treatment occur and could predict OS.

## Conclusion

The present study suggests that the change in serum selenium levels during radiotherapy might be a predictive factor for OS in NSCLC patients. Increasing selenium levels appeared to be associated with improved survival, in particular of metastatic NSCLC patients. As a result, supplementation of selenium in lung cancer patients should be discussed and respective interventional trials require consideration because patients with metastatic disease may benefit from higher selenium levels. In the future, additional independent studies are needed to assess changes in selenium status with or without adjuvant selenium supplementation in larger patient cohorts with respect to survival. This would improve the database regarding the potential importance of selenium in cancer treatment for predicting prognosis during radiotherapy and for testing the potentially beneficial effects of adjuvant supplementation with a cost-effective and safe micronutrient.

## Data Availability

According to the ethics vote, no data can be provided. For anonymized data, further approval from the ethics committee is needed upon request.

## Appendix

**Table 6:** Change in the serum selenium levels of all patients undergoing chemotherapy (*n* = 56) over time

	Univariate analysis			Multivariate analysis														
				Model 1 (I^a^)			Model 2 (II^b^)			Model 3 (III^c^)			Model 4 (IV^d^)			Model 5 (V^e^)		
	HR	95% CI	*p*-value	HR	95% CI	*p*-value	HR	95% CI	*p*-value	HR	95% CI	*p*-value	HR	95% CI	*p*-value	HR	95% CI	*p*-value
t0	0.6	0.2–1.9	0.4	0.7	0.2–2.5	0.6	0.7	0.2–2.6	0.6	0.6	0.2–2.3	0.5	0.4	0.1–1.9	0.3	0.4	0.1–1.8	0.2
t1	0.5	0.3–1.1	0.1	0.5	0.3–1.1	0.1	0.5	0.2–1.0	0.1	0.5	0.2–1.0	0.1	0.7	0.3–1.7	0.5	0.6	0.2–1.5	0.3
t2	0.7	0.4–1.3	0.2	0.7	0.4–1.3	0.2	0.7	0.4–1.3	0.3	0.7	0.4–1.3	0.3	1.1	0.5–2.4	0.8	1.1	0.5–2.4	0.8

^a^I: adjusted for t1/t2

^b^II: adjusted for t1/t2 and age

^c^III: adjusted for t1/t2, age, and sex

^d^IV: adjusted for t1/t2, age, sex, and presence of metastasis (non-metastatic reference, metastatic value)

^e^V: adjusted for t1/t2, age, sex, presence of metastasis (non-metastatic reference, metastatic value), and EQD2

### Initial sample size calculation of the prospective study protocol

The sample size calculation was based on Chen et al. [36]. To determine the sample size, we used the method of Hsieh and Lavori (2000) for Cox proportional hazard models with nonbinary covariates. We assumed a power of 80% and level of significance of 5% (variance = 0.25). A hazard ratio of 2.3 was presumed to be clinically relevant. We considered a survival rate of 20% with a correlation between parameters of 0.7. Thus, we estimated a sample size of 114 patients, which rose to 127 after considering a dropout rate of 10% in each stratum.
